# Mathematical model for evaluating bicarbonate and lactate kinetics in metformin-associated lactic acidosis

**DOI:** 10.1186/s40635-024-00633-8

**Published:** 2024-05-22

**Authors:** João João Mendes, Mauro Pietribiasi

**Affiliations:** 1 Intensive Care Clinic, Faculty of Medicine, University of Lisbon, Avenida Prof. Egas Moniz, 1649-028 Lisbon, Portugal; 2 Nalecz Institute of Biocybernetics and Biomedical Engineering PAS, Księcia Trojdena 4, 02-109 Warsaw, Poland

To the Editor,

Metformin-associated lactic acidosis (MALA) refers to a blood lactate concentration greater than 5 mmol/L and arterial pH less than 7.35 in association with metformin exposure [1]. The mainstay of therapy for MALA is resuscitation and supportive care. Extracorporeal treatments (ECTRs) are indicated if there is failure of standard supportive care, pH ≤ 7.0 or lactate concentration > 20 mmol/L [2].

The use of different ECTRs—conventional hemodialysis (cHD) or continuous renal replacement therapy (CRRT)—is dependent on both clinical factors (e.g., presence of hypotension or shock) and great heterogeneity in equipment availability [3].

There is no mathematical model in the literature describing the complex effect of ECTRs in MALA, which results from the interactions between the delivery of a base (e.g., bicarbonate) via diffusion from a dialysis solution, lactate clearance and metformin clearance.

Using clinical data, we trained a comprehensive, multi-compartment, computational model of carbon dioxide and oxygen transport calculating the acid–base blood biochemistry during hemodialysis [4] to describe the correction of acidosis in MALA. Hyperlactataemia does not mechanistically contribute to acidaemia. Excess hydrogen ions do not result from glycolysis and production of lactate but from other metabolic pathways, namely, the hydrolysis of the ATP generated during anaerobic respiration [5]. We, therefore, described lactate removal by ECTR independently of bicarbonate delivery, using a simple single-pool kinetic model, as lactate clearance does not contribute to pH correction. We then used the models to simulate a comparison of different ECTRs for a virtual patient with MALA. The patient was assumed male, 80 kg, of which 50% as total body water; initial serum bicarbonate concentration was 1.9 mmol/L, pCO_2_ was 17.7 mmHg, and pO_2_ was 142 mmHg (data based on a single case patient admitted in the clinic with MALA).

For cHD, we used: (1) blood flow rate (Qb) of 200 mL/min, the average blood flow obtainable with a conventional provisory catheter; (2) dialysate flow rate (Qd) of 500 mL/min, the normal dialysate flow; and (3) bicarbonate dialysate concentration of 32 mmoL/L, the highest readily available concentration. For CRRT, we considered a standard dose and a high dose scenario. The standard dose scenario corresponds to continuous venovenous hemodialysis (CVVHD) mode with: (1) Qb of 200 mL/min; (2) Qd of 33.3 mL/min, corresponding to an effluent dose of 25 mL/Kg/h which is the maximum standard dose; and (3) bicarbonate dialysate concentration of 32 mmoL/L. The high dose scenario for CVVHD was modelled using Qd of 133.3 mL/min, and the maximum flow rate of the equipment which allows a near-complete saturation of the dialysate. Zero net fluid removal was assumed for all treatments.

The results of the simulation (Fig. 1) demonstrated a greater efficacy of cHD compared to CRRT, which when used should be delivered in maximum dose.

**Fig. 1:**
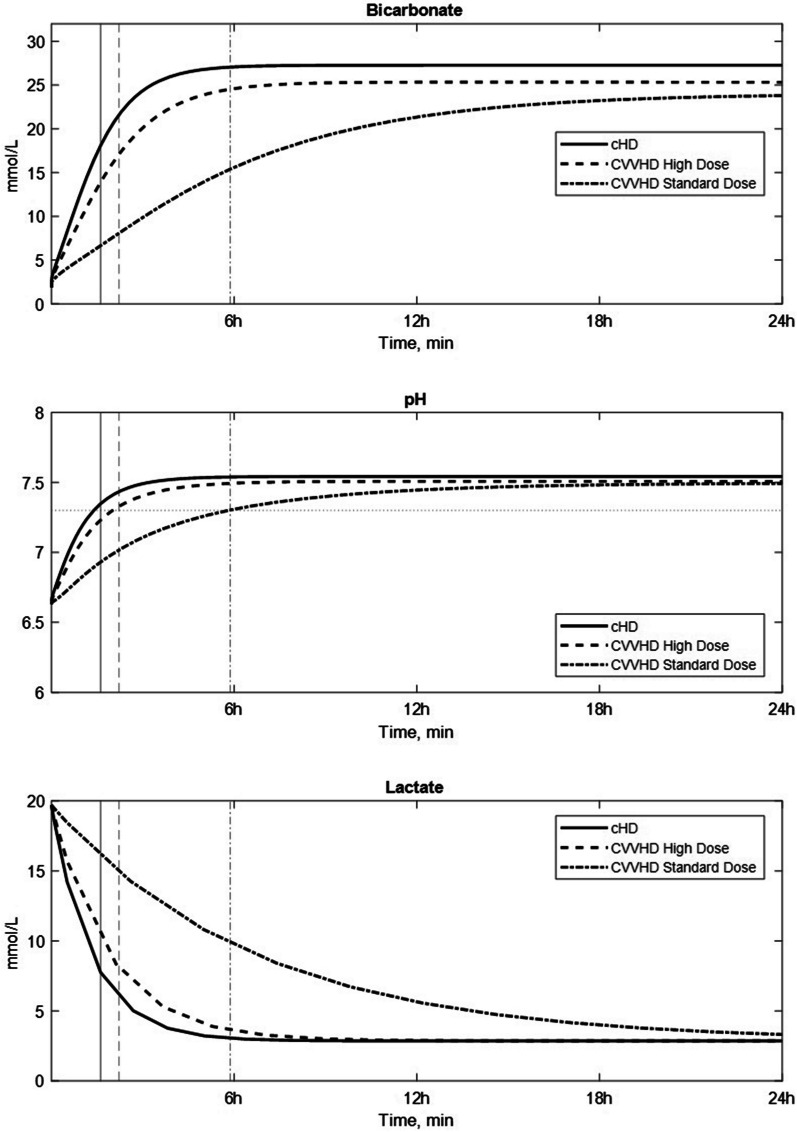
Predicted bicarbonate, pH and lactate concentration profile in the extracellular compartment with conventional hemodialysis (cHD), standard dose continuous venovenous haemodialysis (CVVHD) and high dose continuous venovenous haemodialysis (CVVHD). The vertical lines intersecting the dotted horizontal line represent the instants when the threshold pH of 7.30 is reached (for each treatment, coded as per legend): 97 min for cHD, 133 min for high dose CVVHD and 352 min for standard dose CVVHD)

This simulation provides evidence to support mathematical modelling for comparing the theoretical performance of different ECTRs and help in refining the guidelines for the use of dialysis in treating MALA.

Matlab code: https://doi.org/10.18150/GZRAXA.

## Data Availability

The data that support the findings of this study are available from the corresponding author (JJM) upon request.
